# Toward the sequence-based breeding in legumes in the post-genome sequencing era

**DOI:** 10.1007/s00122-018-3252-x

**Published:** 2018-12-17

**Authors:** Rajeev K. Varshney, Manish K. Pandey, Abhishek Bohra, Vikas K. Singh, Mahendar Thudi, Rachit K. Saxena

**Affiliations:** 1 International Crops Research Institute for the Semi-Arid Tropics (ICRISAT), Hyderabad, 502324 India; 2 ICAR-Indian Institute of Pulses Research (IIPR), Kanpur, 208024 India; 3 International Rice Research Institute (IRRI), IRRI South Asia Hub, ICRISAT, Hyderabad, 502324 India

## Abstract

Efficiency of breeding programs of legume crops such as chickpea, pigeonpea and groundnut has been considerably improved over the past decade through deployment of modern genomic tools and technologies. For instance, next-generation sequencing technologies have facilitated availability of genome sequence assemblies, re-sequencing of several hundred lines, development of HapMaps, high-density genetic maps, a range of marker genotyping platforms and identification of markers associated with a number of agronomic traits in these legume crops. Although marker-assisted backcrossing and marker-assisted selection approaches have been used to develop superior lines in several cases, it is the need of the hour for continuous population improvement after every breeding cycle to accelerate genetic gain in the breeding programs. In this context, we propose a sequence-based breeding approach which includes use of independent or combination of parental selection, enhancing genetic diversity of breeding programs, forward breeding for early generation selection, and genomic selection using sequencing/genotyping technologies. Also, adoption of speed breeding technology by generating 4–6 generations per year will be contributing to accelerate genetic gain. While we see a huge potential of the sequence-based breeding to revolutionize crop improvement programs in these legumes, we anticipate several challenges especially associated with high-quality and precise phenotyping at affordable costs, data analysis and management related to improving breeding operation efficiency. Finally, integration of improved seed systems and better agronomic packages with the development of improved varieties by using sequence-based breeding will ensure higher genetic gains in farmers’ fields.

## Introduction

Grain legumes such as chickpea (*Cicer arietinum*), pigeonpea (*Cajanus cajan*) and groundnut (*Arachis hypogaea*) are highly nutritious crops catering the dietary needs of several hundreds of million people across the world, especially in developing countries (Varshney et al. 2013c; Bohra et al. 2015a). Enormous health benefits associated with these crops earn them the title of “little marvels” (see Considine 2016). Special features of these crops like symbiotic nitrogen fixation and improving soil health encourage inclusion in cropping systems, thus contributing to diversity and sustainability of the system. Sustainable development goals of United Nations seek contribution of grain legume crops particularly concerning malnutrition, income of small-scale food producers and sustainable food production systems (http://gh.one.un.org/content/unct/ghana/en/home/global-agenda-in-ghana/sustainable-development-goals/SDG-2-zero-hunger.html). Although grain legumes are indispensable for global food security and ecosystem resilience, these crops lagged behind cereals in their genetic improvement due to negligence in policy, low investment and lack of genomic resources essential for deploying advanced breeding technologies. Despite these constraints, recognizing the importance of grain legumes in human diet, animal feed and soil health, some efforts were made in the recent past to improve the yield and nutritional quality of legume crops in addition to employing improved agronomy and crop rotation approaches. Indeed, several hundreds of varieties and a few hybrids have been developed in the legume crops using conventional breeding methods for traits such as drought, heat, herbicide, low phosphorus tolerance, early maturity, insect resistance, machine harvestability and high nitrogen fixation (http://www.icrisat.org/impact-of-release-of-256-new-legume-and-119-dryland-cereal-varieties-and-hybrids-in-40-countries-reviewed/). Nevertheless, the rate of genetic gains using the conventional approaches could not bridge the gap between the growing demands (Varshney et al. 2018) which is evident from only 60% increase in pulse production in last 50 years (Foyer et al. 2016).

Low genetic diversity in the breeding programs, lengthy crop breeding cycles, slower adoption of innovative breeding technologies and limited availability of quality seeds to farmers have been the major limitations in delivering higher genetic gains in farmers’ field (Varshney et al. 2018). For instance, 50% of the genetic base of public pigeonpea cultivars is accounted for six founder genotypes (Saxena et al. 2018b). To expedite the breeding cycles, a range of low-to-moderate-scale genomic resources were developed in these crops during the last decade (Varshney et al. 2013c; Pandey et al. 2016). These resources enabled greater understanding of available genetic diversity as well as simplifying complex traits (Varshney et al. 2015). A range of molecular markers (mostly simple sequence repeats, SSRs), bi-parental and natural populations were used for trait dissection and trait improvement (Pandey et al. 2012; Thudi et al. 2014; Varshney 2016). As a result, like cereals, improved lines with enhanced resistance/tolerance to biotic or abiotic stresses, improved agronomic and nutritional traits have been developed in important grain legumes like chickpea and groundnut using marker-assisted backcrossing (MABC) (Pandey et al. 2016; Varshney 2016). Nevertheless, MABC approaches, in general, are successful only for introgression of major effect quantitative trait locus (QTL) or few genes.

The latest sequencing efforts motivated largely by next-generation sequencing (NGS) technologies have culminated in the availability of the reference genomes and re-sequencing of the germplasm and breeding lines in chickpea (Varshney et al. 2013b), pigeonpea (Varshney et al. 2012) and groundnut (Bertioli et al. 2016; Chen et al. 2016). This post-genome sequencing era also witnessed a paradigm shift from marker-based genotyping to sequencing-based genotyping of the breeding populations and diversified germplasm panels. Such efforts have facilitated development of high-density genome/haplotype maps, identification of QTLs and discovery of new genes in several legume crops (Singh et al. 2016a; Pandey et al. 2017b; Singh et al. 2017). Furthermore, deployment of genomic technologies has yielded better breeding outcomes, with a range of molecular breeding products ready for testing and release. This primarily includes targeted improvement in disease resistance (Varshney et al. 2013a, 2014a, b; Kolekar et al. 2017), quality traits (Janila et al. 2016a; Bera et al. 2018) and resilience to changing climatic scenarios (Varshney et al. 2013a).

In the present article, we discuss the technological advances that led grain legumes into the post-genome sequencing era. We provide an update on the molecular breeding products ready for commercial cultivation in these crops. Special emphasis has been placed on potential and challenges in application of sequence-based methods in future breeding programs, and challenges that lie ahead are highlighted.

## Genome sequences

Taking advantage of NGS technologies, draft genomes have been developed for chickpea (Varshney et al. 2013b), pigeonpea (Varshney et al. 2012) and groundnut or peanut (Bertioli et al. 2016; Chen et al. 2016). In chickpea, a high-yielding medium seeded kabuli variety CDC Frontier was used to develop the draft genome comprising 544.73 Mb assembled sequence data representing 73.8% of the total chickpea genome (738.09 Mb) (Varshney et al. 2013b). In the case of pigeonpea, a high-yielding variety Asha (ICPL 87119) was used to generate 237.2 Gb of sequence data. A total of 605.78 Mb could be assembled into scaffolds representing ∼ 73% of pigeonpea genome (Varshney et al. 2012). In groundnut, genome sequences of its diploid ancestors (A and B genomes) were reported. Sequencing of A-genome progenitor, *A. duranensis* (V14167 and PI475845), and B-genome progenitor *A. ipaensis* (K30076) was completed (Bertioli et al. 2016; Chen et al. 2016). A total of 96.0% and 99.2% of the sequence, represented by 1692 and 459 scaffolds, could be ordered into 10 pseudomolecules per genome of 1025 and 1338 Mb for *A. duranensis* and *A. ipaensis*, respectively. Most recently, the high-quality reference genomes were also developed for two subspecies *hypogaea* (https://peanutbase.org/peanut_genome) and *fastigiata* (http://peanutgr.fafu.edu.cn/News_english.php) of cultivated tetraploid. These reference genomes are facilitating comparative and functional genomics studies in addition to genomics-assisted breeding (GAB) in these important legume crops.

### Highlights of three legume draft genomes

The GC contents in all the three mentioned legume genomes were found to be in similar range as 30.78% in chickpea, 32.8% in pigeonpea and 31.79% in groundnut. On the other hand, a number of genes varied significantly in all the three legumes. Within the legumes, pigeonpea and chickpea belong to another sub-classification of pulse crops. These two pulse crops have also shown fold differences in terms of genes identified (48,680 genes in pigeonpea and 28,269 genes in chickpea). In case of groundnut, 50,324 genes (Chen et al. 2016) and 36,734 genes (Bertioli et al. 2016) were identified in *A. duranensis*, while 41,840 genes in *A. ipaensis* (Bertioli et al. 2016). The soon to be available details of tetraploid genome assemblies of groundnut will have more precise and accurate information (personal communications with Scott Jackson, USA; Weijian Zhuang, China; and Xiaoping Chen, China). The huge differences in number of genes identified across three legumes and also within the groundnut genomes may be due to varying quality of the draft genome assemblies as well as different parameters/resources used for gene prediction. We understand that the genome assemblies will be improved with Hi-C based sequencing technology (van Berkum et al. 2010), and then, the huge difference in number of genes may be reduced. The repetitive sequences composed of transposable elements and unclassified repeats in the three legume genomes also varied with 51.67% in pigeonpea, 49.41% in chickpea and 61.7% (*A. duranensis*) to 68.5% (*A. ipaensis*) in groundnut. The reference genomes developed for these three legumes have also been analyzed for crop specific traits, which in turn has facilitated understanding of the genetic variations found in each crop/genotype. For instance, 90 cultivated and wild genotypes from 10 different countries were also re-sequenced in the chickpea draft genome study. Comprehensive analysis of re-sequenced data provided targets of both breeding-associated genetic sweeps and domestication. Further, candidate genes in chickpea genome for disease resistance and agronomic traits have also been identified (Varshney et al. 2013b). Pigeonpea was the second legume crop after soybean and the first non-industrial legume crop with draft genome sequence available in 2012 (Varshney et al. 2012). Draft genome of pigeonpea also provided information on the role of potential gene families in evolution/domestication, e.g., drought tolerance. In groundnut, Bertioli et al. (2016) provided insights into architecture and evolution of subgenomes, genetic exchange between subgenomes and candidate genes for disease resistance, whereas Chen et al. (2016) provided insights into geocarpy, oil biosynthesis and allergens besides providing information about evolution and polyploidization.

### Whole genome re-sequencing

Availability of draft genomes has provided opportunities to deploy whole genome re-sequencing (WGRS)-based approaches in these three legumes. However, it was decided to move forward in a step-wise manner depending on the resources available. Therefore, in these legumes, different sets of genotypes/lines/accessions were selected based on their priority in respective crop improvement programs. As the first step in pigeonpea and chickpea, parents of segregating mapping populations were subjected to WGRS (Kumar et al. 2016; Thudi et al. 2016b). These studies have developed the first-generation HapMaps, signature sequences and large-scale variations for high-resolution trait mapping in pigeonpea and chickpea. Further, reference sets representing diversity present in genetic stocks available in gene bank were targeted for WGRS in pigeonpea (Varshney et al. 2017), chickpea (unpublished) and groundnut (unpublished). In the case of pigeonpea, WGRS data on reference set collection of 292 lines deduced the origin, migration of the crop and identified markers associated with traits of interest for crop improvement (Varshney et al. 2017). In parallel, 104 parental lines of hybrids in pigeonpea (unpublished) and 129 chickpea varieties/elite lines have been sequenced (Thudi et al. 2016a). Detailed analysis of WGRS data on 129 chickpea varieties/elite lines has provided temporal diversity trends across different time zones (Thudi et al. 2016a). Further, the 3000 Chickpea Genome Sequencing Initiative and sequencing of > 100 wild species accessions in pigeonpea have been initiated recently. Approximately 40 lines in groundnut (Pandey et al. 2017a) have also been re-sequenced which include four synthetic tetraploids and their six diploid parents (Chen et al. 2016). Furthermore, sequencing of 300 genotypes of groundnut reference set is underway at ICRISAT for understanding the diversity, and population structure in the germplasm as well as for candidate gene discovery through genome-wide association studies (GWAS).

In summary, the draft genome sequence and re-sequencing data in all three legumes have provided information on genes/genomic segments involved in evolution, domestication, architecture, response to stresses, etc. The WGRS data have also provided access to the unique alleles, signature sequences, and markers and so on for research community. It is important to note that research advances made in these three legumes have also facilitated/motivated/supported other crop species as well. For instance, the pigeonpea genome sequence has been helpful to clone the resistance gene to Asian soybean rust disease that has been only treatable with fungicides in soybean (Kawashima et al. 2016).

## Appropriate genotyping assays for trait mapping and breeding

The sequencing and re-sequencing of diverse germplasm make unlimited genome-wide structural variations available, which facilitate genotyping the genetic and breeding material. A number of marker genotyping platforms from low to high throughput have been deployed during last two decades in crop breeding (Rasheed et al. 2017).

In the case of these legume crops, there is mainly a need of following type of platforms for genetic analysis and breeding applications: (1) high-density genotyping (> 20 K SNPs) of genetic populations for trait mapping using GWAS, (2) medium-density genotyping (2–5 K SNPs) of mapping populations for trait mapping using genetic mapping approach as well as for deploying genomic selection (GS) and (3) low-density genotyping (1–10 SNPs) for performing early generation screening as well as for marker-assisted selection, quality control (QC) and hybrid purity testing. To cater the above-mentioned needs, Axiom^®^ Arrays with more than 50 K SNPs have been developed in chickpea (Roorkiwal et al. 2018a), pigeonpea (Saxena et al. 2018a, b, c) and groundnut (Pandey et al. 2017a). Sequencing-based genotyping approaches such as genotyping-by-sequencing (GBS), RADseq and skim sequencing have also been deployed in these legumes for genetic analysis and breeding applications. Initially, the genomic selection has been performed using GBS and subsequently with the SNP arrays (see Crossa et al. 2017). These analyses indicate that high-density genotyping is not always required for undertaking GS breeding. Therefore, efforts have been started to develop medium-density genotyping assays for uniformly distributed 2000–5000 high informative SNPs for deployment in GS breeding. In this direction, in collaboration with Cornell University and Integrated DNA Technologies company (https://www.idtdna.com/pages/products/qpcr-and-pcr/genotyping/rhamp-snp-genotyping), rhAmp SNP genotyping assays are being developed for about 2000 loci in each of the legume crops.

Regarding low-density genotyping assays, 10-SNP panels have been developed in collaboration with Intertek company for many crop species, including these three legume crops, for performing foreground selection in early generations of breeding program under the high-throughput genotyping project (HTPG) (Varshney 2016, http://cegsb.icrisat.org/high-throughput-genotyping-project-htpg/). This technology is much cheaper as it just costs US$ 1.5–2.0/sample for 10 SNP markers including DNA isolation. This low density or panel of selected genotyping has made task easier for breeding units either located remotely or units without DNA isolation facility. Such small SNP panels are more likely to be developed in future to perform specific tasks such as early generations screening, ensuring seed purity in seed lots and in identifying true F_1_ plants in routine breeding programs.

## Sequencing-based trait mapping

Until 2005, most of these legume crops were facing problems for achieving even low-density genetic mapping due to paucity of polymorphic markers (Pandey et al. 2016; Varshney 2016). Several efforts for trait mapping made in these legume crops could achieve sparsely dense genetic maps, which did not allow genetic mapping at high resolution. Majority of these studies deployed large-scale SSR markers for checking polymorphism among parental genotypes, resulting in identification of limited number of polymorphic SSRs due to low level of polymorphism in cultivated gene pool. After the genome sequencing/re-sequencing data have become available in these legume crops in the last decade, millions of structural variations have been identified which can now be used as genetic markers in trait mapping and breeding. Currently, NGS-based high-throughput genotyping approaches are being deployed in these legumes that offer several advantages over earlier genotyping approaches. The major advantages include time-efficient and faster discovery of genomic regions and candidate genes for downstream applications such as gene cloning and molecular breeding research. The sequencing-based trait mapping can be accomplished by: (i) sequencing all individuals from an experimental population or diversified germplasm panel and (ii) sequencing the pooled samples of extremes phenotypes. These two approaches are now routinely deployed, and several such studies will be completed in coming years (Tables 1 and 2).

**Table 1:** Comparison of pooled and whole genome sequencing-based trait mapping approaches

Description	Pooled based					Whole population based				
	Indel-Seq	Seq-BSA	QTL-seq	MutMap	BSR-seq	WGRS	GBS	SKIM-seq	SNP array	RNA-seq
Cost	Low	Low	Low	Low	Low	High	Medium	Medium	High	Medium
Mapping 1–2 traits at a time	Yes	Yes	Yes	Yes	Yes	No	No	No	No	Yes
Mapping > 2 traits at a time	No	No	No	No	No	Yes	Yes	Yes	Yes	No
DNA/RNA based	DNA	DNA	DNA	DNA	RNA	DNA	DNA	DNA	DNA	RNA
Variants	InDels	SNPs	SNPs	SNPs	SNPs	SNPs	SNPs	SNPs	SNPs	SNPs
Approach	RG = HTP=HTB ≠ LTB	HTP = HTB≠LTB and SNP index	SNP index	SNP frequency	SNPs/FPKM	SNP calls	SNP calls	SNP calls	SNP variation	FPKM
Status of sequencing-based trait mapping in legume crops^†^	+	+	+++	–	–	++	+++	+	+++	–

*RG* reference genome, *HTP* high trait parent, *HTB* high trait bulk, *LTB* low trait bulk, *FPKM* Fragments Per Kilobase of transcript per Million mapped reads

^†^+, ++ and +++ signs indicate technologies utilized in moderate, good and excellent quantity in the legume crops, and – sign indicates technologies yet to be utilized in legume crops

**Table 2:** List of successful sequence-based trait mapping efforts in chickpea, pigeonpea and groundnut

S no.	Sequencing strategy	Trait mapping approach	SNPs used for trait mapping	Target traits	Significant results achieved by these sequencing-based trait mapping approaches	Reference
Chickpea						
1.	GBS of cultivated and wild accessions	Genetic diversity	82,489 SNPs	NIL	Diversity, population genetic structure and linkage disequilibrium (LD)	Bajaj et al. (2015)
2.	GBS of cultivated and wild accessions	Genetic diversity	44,844 SNPs	NIL	Revealed complex admixed domestication pattern, extensive LD estimates (0.54–0.68) and extended LD decay (400–500 kb)	Kujur et al. (2015)
3.	GBS of cultivated accessions	Genetic diversity	3187	NIL	Identified a genetic cluster corresponding to black-seeded genotypes traditionally cultivated in Southern Italy	Pavan et al. (2017)
4.	ddRADseq of biparental population	Genetic mapping	604 bin loci	NIL	Only genetic map could be developed	Deokar et al. (2014)
5.	GBS of biparental population	Genetic mapping	743 SNP loci	Drought tolerance-related traits	Refined the “QTL-hotspot” and region to 14 cM (49 SNPs) and developed cleaved amplified polymorphic sequence (CAPS) and derived CAPS (dCAPS) markers	Jaganathan et al. (2015)
6.	GBS of biparental population	Genetic mapping	3228 SNP loci	Seed traits	Identified 20 QTLs and candidate genes associated with seed traits	Verma et al. (2015)
7.	WGRS of complete biparental population	Genetic mapping	–	Drought tolerance-related traits	Fine-mapped and identified 23 candidate genes by splitting the “QTL-hotspot” region into two subregions, namely “QTL-hotspot_a” (15 genes) and “QTL-hotspot_b” (11 genes)	Kale et al. (2015)
8.	WGRS of pooled samples	QTL-seq	–	100 seed weight	Identified a major genomic region (836,859–872,247 bp) on chromosome 1 and narrowed down the major SWQTL (CaqSW1.1) region into a 35 kb harboring 6 candidate genes for 100 seed weight	Das et al. (2015)
9.	WGRS of pooled samples	QTL-seq	–	100-seed weight and root/total plant dry weight ratio	Identified two significant genomic regions, one on CaLG01 (1.08 Mb) and another on CaLG04 (2.7 Mb) linkage groups for 100SDW. Identified four and five putative candidate genes associated with 100SDW and RTR, respectively. Also two genes (Ca_04364 and Ca_04607) for 100SDW and one gene (Ca_04586) for RTR were validated using CAPS/dCAPS markers	Singh et al. (2016a)
10.	WGRS of pooled samples	QTL-seq	–	Ascochyta blight (AB) resistance	Identified 11 QTLs in CPR-01 and 6 QTLs in CPR-02 populations on chromosomes Ca1, Ca2, Ca4, Ca6 and Ca7. Also identified and validated 6 candidate genes located on chromosomes Ca2 and Ca4 using NGS-based BSA	Deokar et al. (2018)
11.	GBS of cultivated accessions	GWAS	24,620 SNPs	Seed iron and zinc	Identified 16 genomic loci/genes associated with seed-Fe and Zn concentrations	Upadhyaya et al. (2015)
12.	WGRS of cultivated accessions	GWAS	144,000 SNPs	Drought tolerance	Several MTAs significantly associated with yield and yield-related traits under drought-prone environments. Identified four genetic regions containing SNPs significantly associated with several different traits, which was an indication of pleiotropic effects	Li et al. (2018)
*Pigeonpea*						
13.	GBS of three biparental populations	Genetic mapping	484 to 1101 SNPs	Sterility mosaic disease (SMD) resistance	Identified a total of 10 QTLs including three major QTLs across the three populations	Saxena et al. (2017a)
14.	GBS of two biparental populations	Genetic mapping	557 to 1101 SNPs	Fusarium wilt (FW) resistance	Identified 14 QTLs through genetic mapping and three important QTLs up to 56.45% PVE for FW resistance through comparative analysis across populations	Saxena et al. (2017b)
15.	GBS of biparental population	Genetic mapping	306 SNPs	Restoration of fertility (Rf)	Identified region on CcLG08 harboring major QTL up to 28.5% PVE and validated two PCR-based markers	Saxena et al. (2018a)
16.	WGRS of pooled samples	Seq-BSA	–	Fusarium wilt (FW) and sterility mosaic disease (SMD)	Revealed association of four candidate nsSNPs in four genes with FW resistance and four candidate nsSNPs in three genes with SMD resistance. Identified seven candidate SNPs for FW and SMD resistance. Further, in silico protein analysis and expression profiling identified two most promising candidate genes for FW resistance	Singh et al. (2016b)
17.	WGRS of pooled samples	Indel-seq	–	Fusarium wilt (FW) and sterility mosaic disease (SMD)	Identified candidate genomic regions associated with FW and SMD resistance in pigeonpea and detected 16 InDels affecting 26 putative candidate genes. Validation of these 16 candidate InDels revealed a significant association of five InDels (three for FW and two for SMD resistance)	Singh et al. (2017)
18.	WGRS of cultivated and wild accessions	GWAS	Millions of SNPs	Flowering time control, seed development and pod dehiscence.	Deeper insights into diversity, population architecture and domestication in pigeonpea. Identified candidate genes for these traits in pigeonpea have sequence similarity to genes functionally characterized in other plants for flowering time control, seed development and pod dehiscence	Varshney et al. (2017)
*Groundnut*						
19.	ddRADseq of biparental population	Genetic mapping	1621 SNP loci	Late leaf spot (LLS) resistance and plant-type-related traits	Identified small-effect QTLs for LLS and other traits	Zhou et al. (2014) and (2016)
20.	WGRS of complete biparental population	Genetic mapping	8869 SNP loci	Early leaf spot (ELS), late leaf spot (LLS), and Tomato spotted wilt virus (TSWV) resistance	Identified two QTLs for ELS on B05 with 47.42% PVE and B03 with 47.38% PVE, and two QTLs for LLS on A05 with 47.63% and B03 with 34.03% PVE and one QTL for TSWV on B09 with 40.71% PVE. KASP markers were developed for major QTLs and validated in the population	Agarwal et al. (2018)
21.	WGRS of pooled samples	QTL-seq	–	Late leaf spot (LLS) resistance	Identified significant candidate QTLs on three chromosomes, A05, B03, and B05; and developed three KASP markers explaining 15% PVE for LLS resistance	Clevenger et al. (2018)
22.	WGRS of pooled samples	QTL-seq	–	Late leaf spot (LLS) resistance	Identified genomic region on A03 that explains > 80% PVE for rust and > 40% PVE for LLS resistance. Identified 19 candidate genes and validated 6 markers for rust and LLS resistance	Pandey et al. (2017b)

*ddRADseq* double-digest restriction-site-associated DNA sequencing, *GBS* genotyping-by-sequencing, *WGRS* whole genome re-sequencing, *GWAS* genome-wide association study, *Seq-BSA* sequencing-based bulked segregant analysis, *CAPS* cleaved amplified polymorphic sequence, *dCAPS* derived cleaved amplified polymorphic sequence

### Sequencing entire genetic populations

The low-cost sequencing has encouraged researchers for sequencing complete genetic populations such as bi-parental (RIL recombinant inbred line), multi-parents (MAGIC multi-parent advanced generation inter-cross and NAM nested association mapping) and natural populations (different types of diversity panels and training sets). For sequencing an entire population, the sequencing data can be generated with either high coverage (using WGRS) or at low coverage (using GBS and skim sequencing). These genome-wide approaches provide highly informative SNPs at massive scale, which are crucial for high-density genetic mapping, and to facilitate understanding of genetic structures and to perform high-resolution trait mapping.

The sequencing-based genotyping of complete genetic populations or diverse germplasm set has yielded exciting results in several crop species. Notable examples include identification of candidate genomic region/gene and marker-trait-associations through GWAS in rice (Huang et al. 2009). Sequencing-based genotyping has also been undertaken for mapping flowering time control, seed development and pod dehiscence in pigeonpea (Varshney et al. 2017), drought tolerance-related traits in chickpea (Kale et al. 2015) and resistance to early leaf spot, late leaf spot (LLS) and tomato spotted wilt virus (TSWV) in groundnut (Agarwal et al. 2018). For instance, in the case of groundnut, the WGRS of the complete RIL population identified two QTLs for early leaf spot (ELS) on B05 (47.42% PVE) and B03 (47.38% PVE); and two QTLs for LLS resistance on A05 (47.63% PVE) and B03 (34.03% PVE), while one QTL for TSWV resistance on B09 (40.71% PVE) chromosomes (Agarwal et al. 2018). This study also identified candidate genes that were converted into Kompetitive allele-specific PCR (KASP) markers.

In the case of WGRS of diverse germplasm panel, 292 lines of pigeonpea reference set were used for the identification of candidate genes for flowering time control, seed development and pod dehiscence using GWAS approach (Varshney et al. 2017). Similar attempts on re-sequencing of diverse germplasm panel and GWAS are in progress in chickpea and groundnut. For example, the ICRISAT with its partners have made significant progress through “The 3000 Chickpea Genome Sequencing Initiative” (Varshney 2016) in sequencing and further analyzing the data for understanding the population structure and breeding history, and discovery of marker-trait associations and candidate genes for important agronomic traits. More such examples are likely to follow in coming years in these legume crops.

The GBS, another sequencing-based genotyping approach, was deployed in several studies in crops including legumes to discover a large number of genome-wide SNPs for conducting diverse genetic studies such as understanding genetic diversity and population structure, developing high-density genetic maps, QTL analysis, GWAS and genomic selection (GS) (Elshire et al. 2011). The popularity and wider acceptability among researchers for GBS have been due to its cost-effectiveness with the greater scope to implement even in those crops where reference genome is not available. In chickpea, the GBS approach was deployed for studying molecular and genetic diversity, understanding the genetic architecture, population structure and linkage disequilibrium decay in cultivated and wild accessions (Bajaj et al. 2015; Kujur et al. 2015; Pavan et al. 2017). Further, the GBS or double-digest restriction-site-associated DNA sequencing (ddRADseq) facilitated construction of dense genetic maps in chickpea (Deokar et al. 2014; Jaganathan et al. 2015; Kujur et al. 2015; Verma et al. 2015); pigeonpea (Saxena et al. 2017a, b, 2018a) and groundnut (Zhou et al. 2014; unpublished).

An interesting example of use of GBS approach includes saturation of the “*QTL*-*hotspot*” region that harbors QTLs for several drought tolerance relevant traits. This study showed successful narrowing down of the genomic region from 29 to 14 cM (Jaganathan et al. 2015). Similarly, 20 QTLs and candidate genes associated with seed traits were also identified in chickpea using GBS approach in another study (Pavan et al. 2017). In pigeonpea, the GBS-based mapping of two RIL populations led identification of QTLs and candidate genes for resistance to fusarium wilt (FW) and sterility mosaic disease (SMD) (Saxena et al. 2017a, b) in addition to restoration of fertility (Rf) (Saxena et al. 2018a). Similar attempts have been made in groundnut to employ GBS that has led to the identification of major QTLs and candidate genes for foliar disease namely late leaf spot (LLS) and rust resistance (Pandey et al. 2016; unpublished). Deployment of ddRADseq on the other hand yielded mere small-effect QTLs for LLS and other plant-type-related traits in groundnut (Zhou et al. 2016). Additionally, the GBS and skim sequencing approaches have also played key role in improving genome assemblies in chickpea (Ruperao et al. 2014) making them more precise for conducting legume biology and comparative genomics studies (Table 2).

Both above-mentioned approaches, i.e., WGRS and GBS, have limitations and choice of using one approach over the other relies upon the objective of the study (requirement of more vs less markers), nature of genetic material as well as availability of funding resources and technical expertise. For example, the WGRS provides high-quality sequencing data, but it becomes unaffordable if the population is exceptionally large. On the other hand, the GBS generates considerably high number of missing data points across the population at a given locus that may be fine with genetic diversity or linkage mapping but is not deemed suitable for GS breeding. If the genome sequence is not available for a crop, GBS approach can still be used for SNP discovery. However, majority of legume crops including three mentioned legume crops have genome sequence assemblies available. The limitations of both of these approaches partly can be countered by performing sequencing at lower depth, referred as skim sequencing (Golicz et al. 2015). The utility of this approach has been demonstrated in fine mapping of the “*QTL*-*hotspot*” region for drought tolerance-related traits in chickpea (Kale et al. 2015). This study not only delineated the QTL region but also identified 23 candidate genes through resolving the “*QTL*-*hotspot*” region into two subregions namely “*QTL*-*hotspot_a*” (139.22 Kb; 15 genes) and “*QTL*-*hotspot_b*” (153.36 Kb; 11 genes) (Kale et al. 2015).

### Sequencing extreme pools

This latest trait mapping approach relies on sequencing of pooled samples constituted with RILs of extreme phenotype for a given trait that borrows the basic principle of bulked segregant analysis (BSA) (Michelmore et al. 1991). This principle can be applied in different types of bi-parental populations generated either by crossing two contrasting genotypes or by crossing the mutant with the original parent (wild type). Depending on the diverse origin and method of population development, these approaches have been referred to as QTL-Seq (Takagi et al. 2013a), Seq-BSA (Singh et al. 2016a, b), Indel-Seq (Singh et al. 2017), MutMap (Abe et al. 2012), and BSR-Seq (Liu et al. 2012) approaches.

The “QTL-Seq” approach has been deployed in all these three legumes, i.e., in chickpea (Das et al. 2015; Singh et al. 2016a; Deokar et al. 2018), pigeonpea (Singh et al. 2016a) and groundnut (Pandey et al. 2017b; Clevenger et al. 2018). In the case of chickpea, this approach successfully identified a major genomic region (836,859–872,247 bp) on Ca1 chromosome which was then further narrowed down to a 35-kb region harboring six candidate genes for 100 seed weight (Das et al. 2015). Similarly, another such study in chickpea identified two significant genomic regions on Ca1 (1.08 Mb) and Ca4 (2.7 Mb) chromosomes for 100 seed weight (100 SDW) leading to further discovery of four and five putative candidate genes associated with 100SDW and root traits ratio, respectively (Kale et al. 2015). This study also developed and validated CAPS/dCAPS markers for use in molecular breeding. Another research in chickpea identified 17 QTLs from two populations on five chromosomes (Ca1, Ca2, Ca4, Ca6 and Ca7), of which six candidate genes on chromosomes Ca2 and Ca4 were further validated using NGS-based BSA. In case of pigeonpea, this approach was successfully deployed for localization of genomic regions and discovery of candidate genes for days to flowering and obcordate leaf shape (unpublished). In case of groundnut, deployment of QTL-seq approach identified genomic region on A03 chromosome that explains > 80% PVE for rust and > 40% PVE for LLS resistance, followed by the discovery of 19 candidate genes (Pandey et al. 2017b). This study also reported validation of a set of allele-specific markers in breeding populations and germplasm set, thus offering a cost-effective genotyping assay for application in early generation selection. Another study in groundnut was also focused on LLS resistance and identified significant candidate QTLs on three chromosomes, A05, B03, and B05; and three KASP markers were developed that controlled 15% PVE for LLS resistance (Clevenger et al. 2018). Owing to its ability to permit precise and rapid mapping and discovery of candidate genes, QTL-seq approach is most likely to be frequently used across these legumes in coming years for agronomically important traits.

Despite being an effective approach, the QTL-seq approach sometimes does not deliver expected results because of the trait complexity and in such situations, the second pooled sequencing-based approach, namely “Seq-BSA,” can be applied for the identification of candidate SNPs in the targeted genomic regions. This approach calculates genome-wide SNP index of both the extreme bulks using QTL-seq pipeline (Takagi et al. 2013a). Seq-BSA has been successfully utilized for the identification of putative SNPs associated with resistance to FW and SMD in pigeonpea (Singh et al. 2016b). This study revealed association of four candidate nsSNPs in four genes with FW resistance and four candidate nsSNPs in three genes with SMD resistance. Further, this study also reported in silico protein analysis and expression profiling leading to identification of two most promising candidate genes namely *C.cajan_01839* for SMD resistance and *C.cajan_03203* for FW resistance.

The pooled sequencing adopted for QTL-seq and BSA-seq can also be used for performing the third pooled sequencing-based approach, namely “Indel-Seq” which mainly focuses on variations identified in insertions and deletions. This approach was successfully deployed in pigeonpea that identified 16 InDels affecting 26 putative candidate genes associated with resistance to FW and SMD. Validation of these 16 candidate InDels revealed a significant association of five InDels (three for FW and two for SMD resistance) (Singh et al. 2017). The fourth approach called “MutMap” (Abe et al. 2012) facilitates faster discovery of candidate genes from promising EMS-induced mutants. This approach requires crossing of selected mutant plant for a trait with the wild type, which minimizes the background noise in segregating population. This approach has further modifications wherein it avoids developing any population and just uses mutant and wild-type parents for analysis (MutMap+; Fekih et al. 2013) and performing candidate gene discovery in the gap region which could not be sequenced during genome sequencing (MutMap-Gap; Takagi et al. 2013b).

Similarly, the fifth pooled sequencing-based approach, namely “Bulked segregant RNA-Seq (BSR-Seq)” (Liu et al. 2012), uses the RNA in place of DNA and rest of the analysis is performed similar to the BSA-seq using specialized pipeline. Further, the RNA-seq data for pooled samples for extreme phenotypes were analyzed which helped in discovery of candidate gene for grain protein content (GPC) gene *GPC*-*B1* in wheat (Trick et al. 2012). So far these approaches could not be explored in the legume crops. However, we hope to see application of these approaches in some legume crops like groundnut with large and complex genome.

## Molecular breeding product delivery

Although it used to be considered like a dream to see molecular breeding products in these legume crops, especially when we did not have enough number of markers or good genetic maps about 10 years back. However, collaborative efforts across different organizations fueled with power of sequencing and genotyping technologies have made it possible to deliver several molecular breeding products and deployment of genomic technologies in breeding programs.

In the case of chickpea, genotyping-based selections targeting two QTLs (QTL_AR1_ and QTL_AR2_) for *Aschochyta* blight resistance (AB) accounting for 34% and 21% PVE, respectively, led to the development of an advance chickpea line V10 showing marked AB resistance under field conditions (Bouhadida et al. 2013). Consequently, three FW-resistant lines and seven AB-resistant lines were selected from the crosses C 214 × WR 315 and C 214 × ILC 3279, with confirmatory evidence provided by phenotyping (Varshney et al. 2014a). In addition, two more lines, namely Super Annigeri 1 and Improved JG 74 with enhanced resistance race 4 (*foc 4*), have been developed (Mannur et al. 2018). The effectiveness of MAS was also demonstrated through an increase in the frequency of alleles of the markers (CaER and GAA47) associated with AB resistance driven by the phenotypic selection (Castro et al. 2015). More recently, five resistant lines representing *foc2* gene introgressed into the background of Pusa 256 were reported with the help of foreground selection aided by two SSR markers (TA 37 and TA110) (Pratap et al. 2017). Even for a complex trait like drought tolerance, a total of 29 ILs having marked improvement in root traits like rooting depth, root length density and root dry weight were developed within a short span of three years as a result of marker-assisted improvement of the variety JG 11 targeting the “*QTL*-*hotspot*” region (Varshney et al. 2013a). Harboring a number of drought-relevant traits and accounting for up to 58.20% PVE, this “*QTL*-*hotspot*” located on Ca4 offers a robust genomic region for improving drought tolerance in chickpea (Varshney et al. 2014a). This “*QTL*-*hotspot*” is being introgressed in genetic background of several leading and elite varieties at ICRISAT and its collaborating partners.

Markers associated with fertility restoration (Saxena et al. 2018a) and CMS (Sinha et al. 2015) are being used in hybrid pigeonpea breeding programs at ICRISAT. Moreover, a number of markers including SSRs (Saxena et al. 2010; Bohra et al. 2011, 2015b, 2017) and SNPs (unpublished) have been identified/used to facilitate genetic purity testing of pigeonpea hybrids and their parents, thus greatly assisting in hybrid seed production. More recently, a collaborative effort has been initiated by ICRISAT with ICAR-IIPR and other institutions/universities from NARS for accelerated and targeted improvement of ruling mega varieties of pigeonpea in India such as Asha, Maruti, BSMR 736, BSMR 853, PRG 176, UPAS 120, LRG 41, LRG 52, and genotyping-based section is integral part of this initiative.

In groundnut, one SCAR marker was implemented to screen segregating populations and the advanced breeding lines of groundnut for resistance against root-knot nematode [*Meloidogyne arenaria* (Neal)] in USA. A high correlation was observed between the genotyping and nematode resistance phenotype data with up to 6.3% discrepancies, which were possibly due to 5.8% recombination between the resistance gene and the DNA marker (Chu et al. 2007). Application of DNA markers in a backcross breeding program accelerated selection of recombinant progenies carrying nematode resistance and high oleic acid. The selection for nematode resistance was assisted with SCAR, SSR and CAPS markers, while one CAPS marker along with gel-free SNP assay using HybProbe design facilitated selection for high oleic acid. This genotyping-assisted strategy to pyramid nematode resistance with a high O/L trait led to the development of “Tifguard High O/L” within 3 years (Chu et al. 2011). A more recent example of marker-assisted improvement of high oleic acid in groundnut includes MAS- and MABC-based selection of genotypes using allele-specific polymerase chain reaction (AS-PCR) and CAPS markers. This allowed early identification of 82 MABC and 387 MAS derived ILs, with oleic acid level increased up to 1.1 fold and linoleic acid decreasing up to 1.0 fold (Janila et al. 2016a). In another study, recombinant lines of ICGV 05141 showing up to 44% higher oleic acid: linoleic acid ratio was obtained as a result of targeted selection for the alleles *ahfad2a* and *ahfad2b* controlling fatty acids contents in groundnut (Bera et al. 2018) at Directorate of Groundnut Research, India.

In addition to nematode resistance and high oleic acid, the molecular breeding has also been successfully conducted for foliar disease resistance, namely rust and LLS in groundnut. The trait mapping efforts identified candidate QTLs (QTL on A03 explaining up to 82.62% phenotypic variation for rust resistance and up to 67.98% for LLS while one QTL on A02 for LLS resistance with ~ 40% PVE) and genes for these two diseases using SSR-based and sequencing approaches (Khedikar et al. 2010; Sujay et al. 2012; Pandey et al. 2017b). These validated diagnostic markers have been deployed in MABC approach, and three popular varieties (ICGV 91114, JL 24 and TAG 24) of groundnut were improved for resistance to both foliar fungal diseases using the resistance donor, GPBD 4 (Varshney et al. 2014b). Furthermore, multi-location evaluation of these ILs showed up to 79% and 89% gains in pod yield and haulm yield; and early maturity over their respective recurrent parents, in addition to improved resistance level (Janila et al. 2016b). In a similar way, transfer of QTLs for LLS and rust into a susceptible yet popular variety TMV 2 by SSR markers (GM2009, GM2079, GM 2301, GM1839 and IPAHM103) led researchers to achieve two completely homozygous lines (TMG-29 and TMG-46) with improved resistance and 60% yield advantage over the recurrent parent (Kolekar et al. 2017). Many of these molecular breeding lines developed through MABC approach for foliar disease resistance (Varshney et al. 2014b) and high oleic acid (Janila et al. 2016a) are under multi-location testing in All India Co-ordinated Research Project on Groundnut (AICRP-G) for further evaluation and release.

To capture the trait variation that is accounted to QTLs with smaller phenotypic variation, marker-assisted recurrent scheme (MARS) scheme has been proposed that allows assembling of superior alleles of different QTLs in a single genotype or in a breeding population based on crossing of genotypes using marker/QTL information (Eathington et al. 2007). In chickpea, MARS was initiated with elite by elite crosses (JG 11 × ICCV 04112 and JG 130 × ICCV 05107) and informed decisions were reached on mating of genotypes using F_3_ genotyping data and F_5_ phenotyping data. However, much success could not be achieved through this approach in developing promising lines with desired features.

In recent years, GS approach has become popular to introgress several genes with small additive effects in breeding programs. The increasing availability of massive genetic variants on affordable prices coupled with high-quality phenotyping facilities has provided new avenues to implement GS for improving gains from selections/cycle in crop plants. GS improves genetic gains per unit time through facilitating selection of superior individuals from any breeding population without having any phenotypic record (Crossa et al. 2017). In GS, individuals from training population are scored phenotypically and genotypically to estimate GEBVs, and subsequent selections are exercised on the basis of GEBVs. Superiority of GS over phenotyping- and genotyping-based selection models has been established in simulation as well as empirical studies (Eathington et al. 2007; Ziyomo and Bernardo 2013; Cerrudo et al. 2018).

Regarding GS in these three legume crops, only three reports are available so far and all these studies were conducted in chickpea (Roorkiwal et al. 2016, 2018b; Li et al. 2018). Promising results of GS were evident in chickpea from higher prediction accuracies (up to 0.91) obtained for yield-related traits using six different GS models (Roorkiwal et al. 2016). However, low accuracies were observed for seed yield under rainfed environments. In a more recent GS study in chickpea, Li et al. (2018) suggested incorporating information about the significant markers (GWAS results) to different GS models to increase prediction accuracies. As described above, efforts to apply GS in legume crops have been initiated only recently and this may be due to recent development of cost-effective genotyping platforms in these crops. Excellent reviews have been published on GS in plant breeding that assesses various methods/algorithms being used to calculate prediction accuracies (Lorenz et al. 2011; Heslot et al. 2012; Crossa et al. 2017). Implementing GS for crop improvement faces several challenges such as the relatedness between the phenotyped (training sets) and unphenotyped individuals (testing sets), size of the training sets, and type and number of the DNA markers, and importantly, provisions for integrating G × E/marker × environment (M × E) interactions (Nakaya and Isobe 2018; Crossa et al. 2017). As demonstrated in chickpea, the ability of GS to consider multiple variables simultaneously allowed breeding programs to gain higher prediction accuracies through inclusion of G × E effects (Roorkiwal et al. 2018b). This study also reported higher prediction accuracies with DArT Seq system as compared to the GBS. Similar studies on GS have also been initiated in groundnut at ICRISAT. In brief, we expect to have deployment of GS in several breeding programs in legumes.

In addition to GS, the GS + de novo GWAS model combining the RR-BLUP with markers was reported to be promising for enhancing genetic gains in rice. This strategy is expected to fasten the introduction of novel genetic variations in breeding population (Spindel et al. 2016). Furthermore, haplotype-based GWAS and GS would facilitate the rapid identification and utilization of superior versions of target gene(s)/variations, respectively. For instance, a recent GWAS indicated *GmCHX1* as the potential candidate conferring salinity tolerance in soybean. In addition, the genotypes belonging to SV-2 haplotype of *GmCHX1* were found to be highly tolerant, whereas the other two groups, SV-1 &amp; SV-3 groups were sensitive (Patil et al. 2016). Similarly, GWAS and haplotype analysis for grain cooking and eating quality traits in rice resulted in the identification of superior and desired haplotypes associated with the trait (Wang et al. 2017). In near future, haplotype-based GS + *denovo* GWAS will be the promising approach across the crops for targeting superior haplotypes for the development of promising genotypes.

## Harnessing genetic diversity

The urgency to conserve and increase genetic diversity in important food crops is highlighted from the fact that the last century has witnessed a 75% reduction in crop diversity in farmers’ fields, and the climate change is going to reduce it further by nearly 20% by 2050 (see Massawe et al. 2016). In chickpea, a recent study offers evidence of severe domestication bottleneck, with effective population of cultivated chickpea being 100-fold lesser than that of *C. reticulatum* (von Wettberg et al. 2018). Similarly, the genetic diversity of the cultivated chickpea was found to be 100 times lesser than that of wild chickpea (*Cicer reticulatum* and *C. echinospermum*). Analysis of landraces and wild relatives in legume crops is greatly assisted by current advances in genomics, phenotyping and computational biology. Analysis of 147 chickpea landraces, housed at the Vavilov Institute of Plant Genetic Resources (VIR), Russia, elucidated genomic basis of a set of “human-selected adaptations.” Importantly, these landraces were collected from Turkey and Ethiopia, which represent center of origin and center of diversity, respectively, of chickpea. Combining high-density genotyping data with the historical phenotypic records on these VIR landraces enabled access to “agro islands” or “domestication islands” in chickpea genomes that show significant associations with multiple phenotypes (Plekhanova et al. 2017). Such “genomic gems” containing co-adapted and co-localized gene complexes have also been reported in chickpea on LG4 and LG2 containing multiple genes/QTLs associated with drought and disease resistance, respectively. Earlier, WGRS/RADSeq of 90 *Cicer* accessions including cultigens, landraces and wild accessions uncovered a large set of 54 genes on LG3 that possibly has been targeted during modern breeding efforts for manipulation of important traits like flowering time (Varshney et al. 2013c).

Similarly in pigeonpea, a genomic region having abundance of MTAs for agronomically important traits was detected on LG9 following re-sequencing of 292 accessions (Varshney et al. 2017). In addition, phylogenetic relationships inferred from WGRS data allowed authors to identify *C. cajanifolius* accession ICP15629, a possible early domesticate with greater agronomic suitability for crop production (Varshney et al. 2017). Accessibility to such genomic “islands” helps in defining “breeding targets” to reintroduce genetic diversity that is lost in modern breeding programs as a consequence of domestication and crop improvement. In view of the escalating problem of habitat loss/degradation, urbanization and shifting land use, systematic efforts are needed to conserve and characterize the germplasm collections that span genetic and geographic breadth. Attempts by Khoury et al. (2015) in this regard are noteworthy with authors analyzing pigeonpea ex situ conservations, identifying the high priority crop wild relatives (CVRs) for further collection and finally, highlighting CWRs with potential traits for use in abiotic stress breeding. A recent survey of chickpea germplasm from “Fertile Crescent” informed by GIS technology led authors to initiative a large-scale introgression breeding program in order to archive broad based genetic populations that harbor potential traits that could facilitate chickpea improvement (von Wettberg et al. 2018). Judicious exploitation of landraces and wild relatives to devise solutions for future needs warrants not only understanding domestication patterns and evolutionary history but also conserving CWRs and accelerating their deployment in pre-breeding programs. Pre-breeding programs will be greatly benefited from GS models that help in prioritizing the accessions for subsequent introgression (Crossa et al. 2017).

In case of groundnut, although the genus *Arachis* is blessed with enormous genetic variability with 79 wild species and cultivated peanut, the crop faces huge challenge because of the differences in ploidy levels in different species which create big genetic barrier in exchanging the genetic diversity (Sharma et al. 2017). Even if some diploid species have cross-compatibility with the tetraploid species, it requires several generations of selfing for selecting desirable tetraploid recombinants. Therefore, the only solution to this problem is development of synthetic groundnut by doubling the chromosome number of the hybrid derived from two diploid species (Simpson et al. 1993; Mallikarjuna et al. 2011). Once these synthetic groundnuts are developed, then these can be evaluated for traits of interest and crosses can be made for transferring useful genes/alleles into cultivated genetic backgrounds. Successful examples are already available in transferring useful traits such as resistance nematode, late leaf spot and rust resistance and other yield component traits in groundnut (Simpson et al. 2003; Kumari et al. 2014; Sharma et al. 2017; Khera et al. 2018). Nevertheless, large-scale and dedicated efforts are required for generating diverse pre-breeding material from different sources so that the primary gene-pool of groundnut can be enriched with desirable and useful alleles.

## Adopting speed breeding to accelerate genetic gains

Genomics-assisted breeding improves genetic gains (ΔG) through enhancing diversity (σ^2^P), favorable gene action (h^2^) and intensity of selection (i) (Moose and Mumm 2008). Likewise, genetic gains can also be improved by shortening the breeding cycles (L) and this rapid generation advance (RGA) can be achieved through controlling temperature, photoperiod, humidity, and harvesting and germination of immature seeds (Watson et al. 2018; Chiurugwi et al. 2018). Among legume crops, the potential of speeding breeding in reducing generation time in breeding programs has been demonstrated in chickpea (Gaur et al. 2007; Watson et al. 2018), pigeonpea (Saxena et al. 2018a, b, c), and groundnut (O’Connor et al. 2013). In chickpea, 4–6 generations could be obtained within a year with plant growth accelerated with extended photoperiod of 22 h and a temperature regime of 22/17 °C (Watson et al. 2018). Earlier, early flowering in chickpea was induced following a 24-hr photoperiod, which enabled production of three generations per year with the help of off-season nursery (Gaur et al. 2007). Similarly, the RGA technology in pigeonpea facilitated accelerated production of 3–4 generations per year with 100% germination reported from immature seeds harvested from 35-day-old plants (Saxena et al. 2017a, b). In groundnut, by integrating speed breeding with single seed descent (SSD) method, O’Connor and colleagues achieved the *F*_2_ to *F*_5_ progression within 17 months in greenhouse settings (controlled environment, 24-h photoperiod and optimal temperatures) in comparison with 42 months invested in achieve the same with field-oriented pedigree breeding method. Under green house facility, the emergence rates were found to be 94% and 91% in *F*_2_ and *F*_3_, respectively, with the corresponding seed recovery rates being 68% and 74%. Speed breeding in legumes crops is in infancy stage, and the major challenges hampering large-scale adoption of speed breeding in these crops include poor access to the infrastructure including electricity-controlled environment and the lack of trained/skilled personnel to operate the facility. As suggested by Chiurugwi et al. (2018), these challenges can be met to a great extent with the development of transportable custom-designed chambers or establishment of speed breeding centers at institutes that could lend these services to smaller and resource-poor breeding programs. Similarly, integration of speed breeding with genomics-assisted breeding and high-throughput phenotyping systems will make the cost associated with speed breeding reasonable. However, most critical to this adoption will be identification of the plant growth phases, plant traits and the protocols that help in maximizing the benefits accruing from speed breeding in orphan crops like legumes.

## Toward the sequence-based breeding

**Fig. 1:**
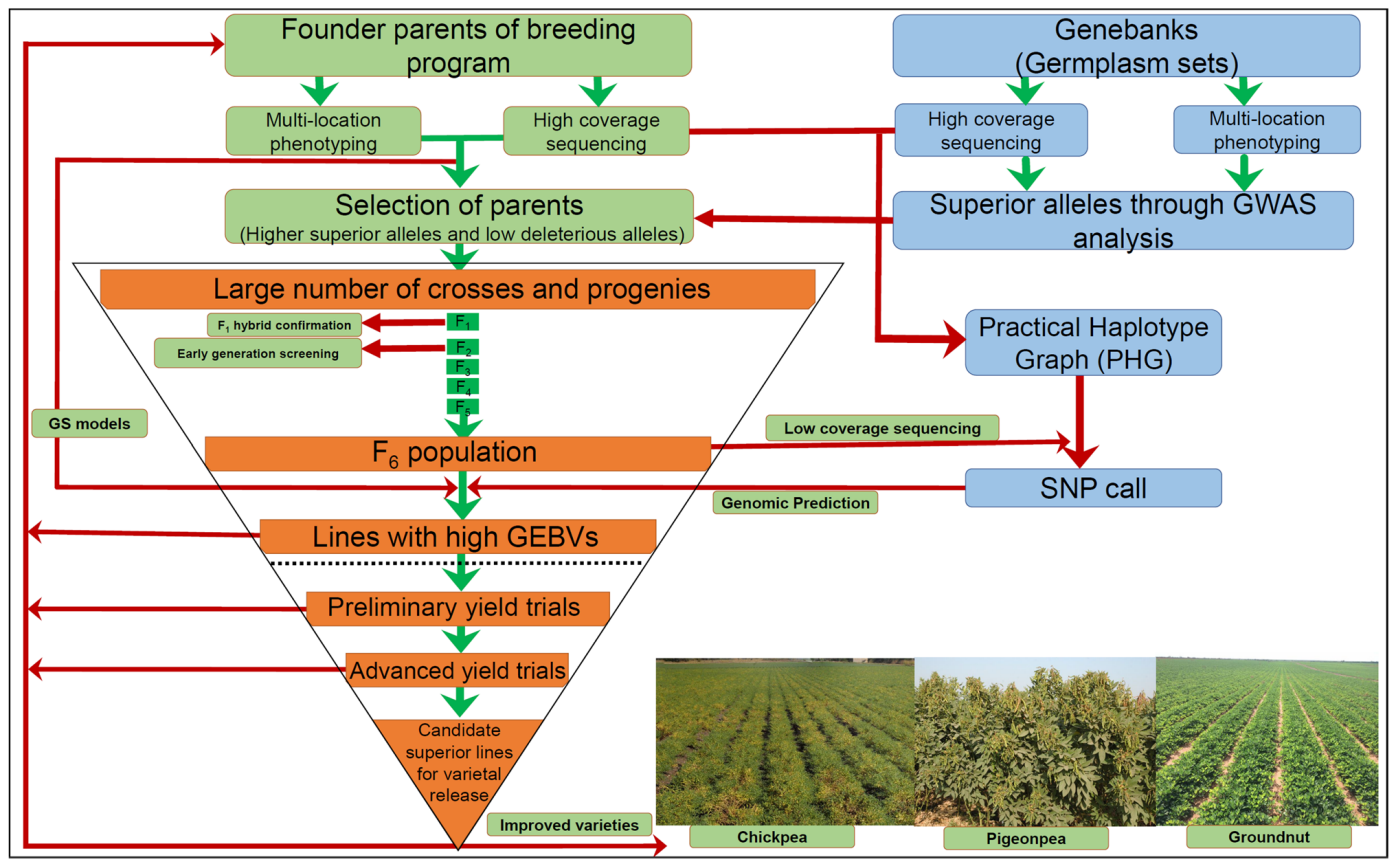
Flowchart for developing improved legume varieties through sequence-based breeding. The next-generation genomic tools and high-throughput phenotyping systems enable harnessing the superior alleles harbored within vast genetic resources of grain legume crops. Genotyping-based techniques have yielded promising results with the delivery of a variety of molecular breeding products in these crops. In this post genomics era, a shift from genotyping- to sequencing-based assays coupled with our enhanced capacity to integrate multi-omics science or a systems biology approach promises to accelerate the genetic gains. Breeder’s decisions greatly informed by such modern advances will reflect in higher productivity gains with fewer resources and in less time

While molecular breeding products have been developed using MAS/MABC in major and some legume crops, these approaches are suitable to introgress 1–3 gene combinations. However, stacking large number of genes in one genetic background through backcross or assembling all the genes through forward breeding (early generation screening) utilizing low-cost genotyping system remains a challenging task. For instance, for targeting eight genes, a total of 256 distinct types of *F*_1_ gametes (2^*n*^, n is the number of target genes), 6561 different genotypes in *F*_2_ population (3^*n*^, n is the number of target genes) and 65,536 would be the size of the smallest perfect *F*_2_ population (4*n*, *n* is the number of target gene). Genotyping such a large number of individuals with low-throughput marker systems like SSR would be labor-intensive and cost-inefficient, and more importantly, decision for selection of lines for making crosses may not be made prior to the initiation of flowering. Although analyzing a large number of advanced lines in any of the breeding programs is possible with genotyping, the higher costs associated with genotyping may force breeding programs to reduce the number of lines for genotyping. This will, in turn, affect the selection intensity. In addition to above, it is also important to mention that MABC/MAS are good approaches for introgressing one or few traits for which leading/elite/mega varieties have become deficient. However, these approaches are not essentially meant for accelerating genetic gains. In our view, the breeding programs in the post-genome sequencing era need to emphasize more on continuous population improvement as compared to improve few varieties. In this context, sequence-based approaches may be very helpful to integrate into the breeding programs (Fig. 1). A tentative outline of sequence-based breeding is given below.

In the first instance, all possible parental lines for a given breeding program need to be sequenced, if possible, at higher depth. With the assumption of the availability of phenotyping data on these lines for a number of years, if possible, approaches like GWAS or available HapMap based on sequencing of founder genotypes can be used to select suitable parental combinations with higher frequency of superior alleles and with limited number of deleterious alleles. After making significantly large number of crosses with higher number of lines, early generation screening can be made with existing 10 SNP panels for a given crop. Selected lines from such crosses can be subjected for GS analysis by using the training model developed on the germplasm set representatives of the segregating populations. For GS, the best genotyping platform is the fixed SNP array that provides high-quality genotyping data and requires minimal analytical skills. However, from the cost perspectives, fixed SNP arrays-based genotyping may not be affordable to large-scale breeding programs. In fact, we propose to sequence these segregating progenies at F6/F7 generations at lower coverage like skim sequencing or 384-plex-based GBS-based genotyping so that cost of sequencing per line remains minimal. If this cost is not affordable at present, we assume that this is going to happen very soon. Till that time, we would like to propose use of practical haplotype graph and capture-sequencing of a fixed number of SNP loci on the segregating populations. In brief, sequencing of parental lines, as well as the sequencing data of the other available germplasm lines, can be used for developing practical haplotype graph (PHG) (https://bitbucket.org/bucklerlab/practicalhaplotypegraph/wiki/Home). Based on this PHG, 2000-5000 SNP loci, depending on the requirement for deployment of GS in a given crop, can be selected. Subsequently, by using any sequence-based approach, these SNP loci can be assayed using rhAmp (Integrated DNA Technology) or DArTseqLD (https://www.diversityarrays.com/index.php/technology-and-resources/dartseq/) technologies. Subsequently based on these data, GS breeding can be deployed on these segregating populations and superior lines with higher GEBVs can be selected. While several of these lines based on preliminary yield trials and advanced yield trials at multiple locations can be taken out for possible candidates for varietal development or use as parent for hybrid breeding programs (e.g., in pigeonpea), we strongly propose use of the best lines from here for integrating in the crossing programs so that next round of breeding populations will be better than the previous round of the populations. We believe by using the sequence-based breeding approach, it will be possible for continuous improvement of populations and accelerate genetic gains at the end of each breeding cycle. This scheme is suitable not only for the legume crops mentioned in this article but in general for all the crops.

## Summary and outlook

Legume crops such as chickpea, pigeonpea and groundnut, though keys for food and nutritional security as well as environmental sustainability, have been lagging as compared to major cereal crops in terms of integration of genomic technologies in breeding programs. These crops have achieved now optimum genomic resources required for the faster trait discovery and accelerated breeding. We believe that while MAS/MABC can be continued to improve elite/mega varieties for a few traits, parental selection, early generation screening and GS, ideally all in combination or combination of one and another or even independently should become the integral part of breeding programs. While sequence-based genotyping approaches are already affordable for several applications, several of these approaches or some new approaches will be available in coming future in such a manner that it will be possible to generate genome information in part or full at the rate of US$ 5 per sample (including DNA extraction). This cost is much cheaper than multi-location evaluation of a line in 2–3 replicates. However, the question is if we will be able to access high-quality and precise phenotyping data at lower cost that is required for trait discovery? Furthermore, do we have standardized phenotyping protocols to score phenotypes for several complex traits in a precise, accurate, high-throughput and cost-effective manner? Similarly, while we are proposing the sequence-based breeding approach here and it is possible to generate genome information for segregating populations in affordable costs, if not now, then very soon, what about data analytical skills in the public breeding programs especially in developing countries? Are our breeding programs well equipped with appropriate field experiment design, bar-code-based labeling of plots, handheld-based data recording and databases containing genotyping and phenotyping? While we may not have answers and solutions for all these questions, we are optimistic that several of these challenges will be addressed in coming years, especially due to several multi-institutional initiatives such as Excellence in Breeding Platform (http://excellenceinbreeding.org/) and Integrated Breeding Platform (https://www.integratedbreeding.net/).

Having realized above, we are hopeful that the sequence-based breeding approach will come of the age soon. While this approach should be able to help breeding programs for developing faster and superior varieties, it is important that these varieties should reach farmers in reasonable time. In this regard, we need to improve seed delivery system in developing countries (Varshney et al. 2018). Furthermore, farmers need to be trained to adopt appropriate agronomy practices together with better seeds of improved varieties so that they can have higher productivity. Finally, the value chain needs one more step and that is providing farmers’ access to markets through digital technologies so that farmers do not just produce more but also can earn more. In summary, we see a huge potential of integration of genomic technologies together with other innovations like speed breeding to accelerate genetic gains not only in breeding and research plots but also to deliver in farmers’ fields.

### Author contribution statement

RKV conceptualized idea, planned MS content and finalized the MS. MKP planned the MS content, coordinated with other co-authors, contributed special sections, brought flow between different sections, and finalized/revised the MS. AB, VKS, MT and RKS planned the MS and contributed special sections.
